# *N*,*N*-Bis(pyridin-2-ylmeth­yl)cyclo­hexa­namine

**DOI:** 10.1107/S1600536812027572

**Published:** 2012-06-23

**Authors:** Matthew P. Akerman, Mathias Chipangura, Allen Mambanda, Deogratius Jaganyi

**Affiliations:** a School of Chemistry and Physics, University of KwaZulu-Natal, Private Bag X01, Scottsville 3209, Pietermaritzburg, South Africa

## Abstract

The pyridine rings of the title compound, C_18_H_23_N_3_, are in a nearly perpendicular orientation relative to the plane defined by the three amino-bonded C atoms, making dihedral angles of 87.4 (1) ° and 84.2 (1) °. One of the pyridine N atoms acts as an hydrogen-bond acceptor for two pyridine C—H groups. By means of these intermolecular hydrogen bonds, the mol­ecules form a two-dimensional network parallel to the *ab* plane.

## Related literature

For a kinetic and mechanistic study of the platinum(II) chelate of the title compound, see: Mambanda & Jaganyi (2012 ▶). For the synthesis of the title compound, see: Sato *et al.* (1992 ▶); Toftlund & Yde-Andersen (1981 ▶); Anderegg & Wenk (1967 ▶). For the crystal structure of the related compound *N*,*N*-bis­(2-pyridyl­meth­yl)-*tert*-butyl­amine, see: Mambanda *et al.* (2009 ▶). For the crystal structures of the hexa­dentate analogues, see: Mambanda *et al.* (2007 ▶). For dinuclear platinum(II) complexes structurally related to the complex of the title compound, see: Hofmann & van Eldik (2003 ▶); Erteurk *et al.* (2007 ▶, 2008 ▶). For dinuclear metal complexes containing bis­(tridentate) chelates structurally related to the title compound, see: Fujihara *et al.* (2004 ▶); Gunatilleke & Norman (2003 ▶); Fujii *et al.* (2003 ▶). For manganese–oxo complexes of *N*,*N*-bis­(2-pyridyl­meth­yl)ethyl­amine and *N*,*N*-bis­ (2-pyridyl­meth­yl)-*tert*-butyl­amine, see: Pal *et al.* (1992 ▶) and Mok *et al.* (1997 ▶), respectively.
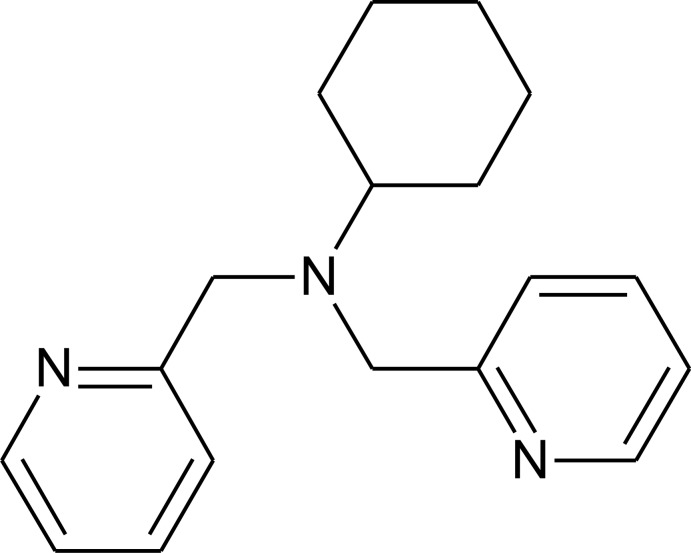


## Experimental

### 

#### Crystal data

C_18_H_23_N_3_
*M* *_r_* = 281.39
Monoclinic, 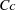

*a* = 6.2272 (2) Å
*b* = 18.1729 (7) Å
*c* = 14.3213 (5) Å
β = 102.118 (4)°
*V* = 1584.57 (10) Å^3^
*Z* = 4
Mo *K*α radiation
μ = 0.07 mm^−1^
*T* = 120 K
0.60 × 0.50 × 0.30 mm

#### Data collection

Oxford Diffraction Xcalibur 2 CCD diffractometer
Absorption correction: multi-scan (Blessing, 1995 ▶) *T*_min_ = 0.959, *T*_max_ = 0.979
7854 measured reflections
2521 independent reflections
2134 reflections with *I* > 2σ(*I*)
*R*_int_ = 0.033

#### Refinement

*R*[*F* ^2^ > 2σ(*F* ^2^)] = 0.038
*wR*(*F* ^2^) = 0.085
*S* = 0.98
2134 reflections
190 parameters
2 restraints
H-atom parameters constrained
Δρ_max_ = 0.15 e Å^−3^
Δρ_min_ = −0.25 e Å^−3^

### 

Data collection: *CrysAlis CCD* (Oxford Diffraction, 2008 ▶); cell refinement: *CrysAlis CCD*; data reduction: *CrysAlis RED* (Oxford Diffraction, 2008 ▶); program(s) used to solve structure: *SHELXS97* (Sheldrick, 2008 ▶); program(s) used to refine structure: *SHELXL97* (Sheldrick, 2008 ▶); molecular graphics: *WinGX* (Farrugia, 1999 ▶); software used to prepare material for publication: *publCIF* (Westrip, 2010 ▶).

## Supplementary Material

Crystal structure: contains datablock(s) I, global. DOI: 10.1107/S1600536812027572/ld2063sup1.cif

Structure factors: contains datablock(s) I. DOI: 10.1107/S1600536812027572/ld2063Isup2.hkl

Supplementary material file. DOI: 10.1107/S1600536812027572/ld2063Isup3.cml

Additional supplementary materials:  crystallographic information; 3D view; checkCIF report

## Figures and Tables

**Table 1:** Hydrogen-bond geometry (Å, °)

*D*—H⋯*A*	*D*—H	H⋯*A*	*D*⋯*A*	*D*—H⋯*A*
C4—H4⋯N2^i^	0.95	2.55	3.475 (2)	166 (1)
C11—H11⋯N2^ii^	0.95	2.64	3.511 (2)	153 (1)

Symmetry codes: (i) 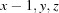; (ii) 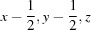.

**Fig. 1.**
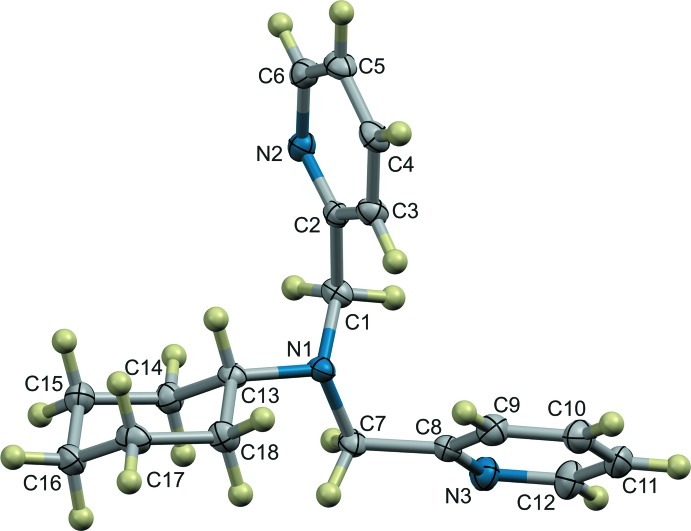
Thermal ellipsoid plot of (1), rendered at 50% probability. Hydrogen atoms are shown as spheres of arbitrary radius.

**Fig. 2.**
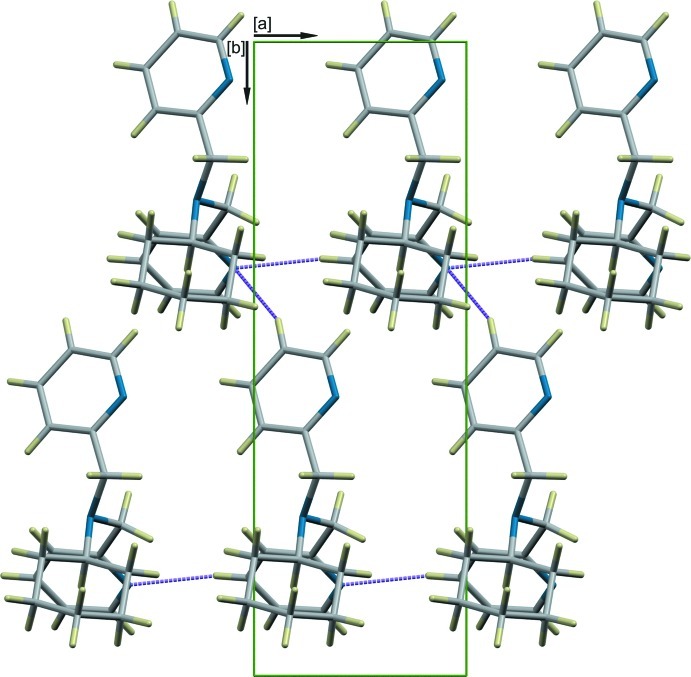
Two-dimensional hydrogen-bonded network of 1 viewed along the c-axis. Hydrogen bonds are represented by dashed purple lines.

## supplementary crystallographic information

### Comment

The search for chelating ligands for the coordination of platinum(II) ions has led us to investigate bis-*N*-functionalized cyclohexylamine as a potential tridentate *N*-donor ligand.

The pyridine rings are in a near perpendicular orientation relative to the three-atom mean plane defined by the N-bonded carbon atoms making angles of 87.4 (1) ° and 84.2 (1) ° to the plane, for the rings containg N2 and N3, respectively. The near perpendicular orientation of the pyridyl rings allows for hydrogen bonding, stabilizing the lattice.

There are non-classical hydrogen bonds between the pyridine nitrogen atom, N2, and the pyridine hydrogen atoms, H4 and H11 of two separate, adjacent molecules. N2 acts as an acceptor for both hydrogen bonds. These bonds lead to the formation of an infinite two-dimensional hydrogen-bonded network. This network is co-planar with the *ab* plane. The network consists of one-dimensional chains with adjacent molecules linked by the N2···H4 hydrogen bond. These one-dimensional chains are then cross-linked by the N2···H11 hydrogen bond, thus forming an infinite, two-dimensional network. Although hydrogen bond length does not necessarily correlate linearly to bond strength, due to packing constraints in the lattice, these bonds are considerably shorter than the sum of their van der Waals radii and are thus likely to be moderate to high in strength. This also seems likely as the D—H···A bond angle of both bonds, 165.7 (1) ° and 153.1 (1) ° for N2···H4—C4 and N2···H11—C11 respectively, do not show a marked deviation from ideality. The hydrogen bond lengths and angles are summarized in Table 1.

### Experimental

The tridentate ligand is formed by reacting two molar equivalents of 2-picolyl chloride hydrochloride under basic aqueous conditions with one molar equivalent of cyclohexylamine, following an improved method of Sato *et al.*, (1992) previously reported by Toftlund & Yde-Andersen (1981) as well as Anderegg & Wenk (1967). Colourless crystals were obtained by slow evaporation of an ethanol solution of the ligand over a period of several days. Yield: 1.276 g (44%).

### Refinement

The positions of all C-bonded hydrogen atoms were calculated using the standard riding model of *SHELXL97* (Sheldrick, 2008) with C—H(aromatic) distances of 0.95 Å and *U*_iso_ = 1.2 *U*_eq_, *C*—*H*(methylene) distances of 0.99 Å and *U*_iso_ = 1.2 *U*_eq_ and a C—H(methine) distance of 1.00 Å and *U*_iso_ = 1.2 *U*_eq_. In the absence of significant anamalous scattering, Friedel pairs were merged.

### Crystal data

**Table:**

C_18_H_23_N_3_	*F*(000) = 608
*M**_r_* = 281.39	*D*_x_ = 1.180 Mg m^−^^3^
Monoclinic, *C**c*	Mo *K*α radiation, λ = 0.71073 Å
Hall symbol: C -2yc	Cell parameters from 2134 reflections
*a* = 6.2272 (2) Å	θ = 3.5–32.1°
*b* = 18.1729 (7) Å	µ = 0.07 mm^−^^1^
*c* = 14.3213 (5) Å	*T* = 120 K
β = 102.118 (4)°	Planar, colourless
*V* = 1584.57 (10) Å^3^	0.60 × 0.50 × 0.30 mm
*Z* = 4	

### Data collection

**Table:**

Oxford Diffraction Xcalibur 2 CCD diffractometer	2521 independent reflections
Radiation source: fine-focus sealed tube	2134 reflections with *I* > 2σ(*I*)
Graphite monochromator	*R*_int_ = 0.033
ω scans at fixed θ angles	θ_max_ = 32.1°, θ_min_ = 3.5°
Absorption correction: multi-scan (Blessing, 1995)	*h* = −9→6
*T*_min_ = 0.959, *T*_max_ = 0.979	*k* = −26→25
7854 measured reflections	*l* = −20→20

### Refinement

**Table:**

Refinement on *F*^2^	Primary atom site location: structure-invariant direct methods
Least-squares matrix: full	Secondary atom site location: difference Fourier map
*R*[*F*^2^ > 2σ(*F*^2^)] = 0.038	Hydrogen site location: inferred from neighbouring sites
*wR*(*F*^2^) = 0.085	H-atom parameters constrained
*S* = 0.98	*w* = 1/[σ^2^(*F*_o_^2^) + (0.053*P*)^2^] where *P* = (*F*_o_^2^ + 2*F*_c_^2^)/3
2134 reflections	(Δ/σ)_max_ < 0.001
190 parameters	Δρ_max_ = 0.15 e Å^−^^3^
2 restraints	Δρ_min_ = −0.25 e Å^−^^3^

### Special details

**Table:**

Experimental. Yield: 1.3432 g (40%), colourless block crystals. ^1^H NMR (400 MHz, CDCl_3_) δ (p.p.m.): 8.58 (d, 2H); 8.50–7.60 (m, 4H); 7.05 (t, 2H); 3.39 (s, 4H); 2.55 (m, 1H); 1.90 (d, 2H); 1.8 (m, 2H); 1.60 (d, 2H); 1.35 (m, 2H); 1.19 (m, 2H). ^13^C NMR (100 MHz, CDCl_3_) δ / p.p.m.: 27.0; 29; 57.0; 60.5; 122.0; 123.0; 136.0; 148.0; 161. IR (KBr, 4000–400 cm^-1^): 2958–2854 (alkyl C—H stretch); 1589 C=N (pyridyl). MS—ES+, m/e: 282.2069, (*M* +1)+. Anal. Calc. for C_18_H_23_N_3_: C, 76.81; H, 8.24; N, 14.93; Found: C, 76.8; H, 8.18; N, 14.89.
Geometry. All s.u.'s (except the s.u. in the dihedral angle between two l.s. planes) are estimated using the full covariance matrix. The cell s.u.'s are taken into account individually in the estimation of s.u.'s in distances, angles and torsion angles; correlations between s.u.'s in cell parameters are only used when they are defined by crystal symmetry. An approximate (isotropic) treatment of cell s.u.'s is used for estimating s.u.'s involving l.s. planes.
Refinement. Refinement of *F*^2^ against ALL reflections. The weighted *R*-factor *wR* and goodness of fit *S* are based on *F*^2^, conventional *R*-factors *R* are based on *F*, with *F* set to zero for negative *F*^2^. The threshold expression of *F*^2^ > 2σ(*F*^2^) is used only for calculating *R*-factors(gt) *etc*. and is not relevant to the choice of reflections for refinement. *R*-factors based on *F*^2^ are statistically about twice as large as those based on *F*, and *R*- factors based on ALL data will be even larger.

### Fractional atomic coordinates and isotropic or equivalent isotropic displacement parameters (Å^2^)

**Table:**

	*x*	*y*	*z*	*U*_iso_*/*U*_eq_	
C1	0.8715 (2)	0.26180 (9)	0.20552 (11)	0.0220 (3)	
H1A	1.0103	0.2846	0.1967	0.026*	
H1B	0.9082	0.2135	0.2369	0.026*	
C2	0.7709 (2)	0.31007 (8)	0.27025 (10)	0.0186 (3)	
C3	0.5510 (2)	0.30374 (9)	0.27499 (11)	0.0235 (3)	
H3	0.4570	0.2709	0.2338	0.028*	
C4	0.4709 (3)	0.34586 (10)	0.34038 (12)	0.0258 (3)	
H4	0.3215	0.3420	0.3451	0.031*	
C5	0.6111 (3)	0.39369 (10)	0.39884 (12)	0.0265 (3)	
H5	0.5610	0.4230	0.4449	0.032*	
C6	0.8260 (3)	0.39748 (9)	0.38825 (12)	0.0253 (3)	
H6	0.9221	0.4308	0.4278	0.030*	
C7	0.7975 (3)	0.18410 (9)	0.06711 (11)	0.0219 (3)	
H7A	0.9583	0.1856	0.0719	0.026*	
H7B	0.7258	0.1839	−0.0015	0.026*	
C8	0.7384 (3)	0.11434 (8)	0.11310 (11)	0.0215 (3)	
C9	0.5374 (3)	0.10687 (10)	0.14035 (12)	0.0270 (3)	
H9	0.4348	0.1463	0.1310	0.032*	
C10	0.4884 (3)	0.04184 (10)	0.18101 (13)	0.0310 (4)	
H10	0.3523	0.0358	0.2002	0.037*	
C11	0.6412 (3)	−0.01444 (10)	0.19330 (13)	0.0313 (4)	
H11	0.6137	−0.0598	0.2216	0.038*	
C12	0.8340 (3)	−0.00279 (10)	0.16339 (14)	0.0321 (4)	
H12	0.9380	−0.0417	0.1713	0.039*	
C13	0.7079 (2)	0.31684 (8)	0.05181 (11)	0.0196 (3)	
H13	0.6855	0.3590	0.0936	0.024*	
C14	0.9088 (3)	0.33578 (9)	0.01086 (12)	0.0240 (3)	
H14A	1.0392	0.3406	0.0636	0.029*	
H14B	0.9370	0.2955	−0.0315	0.029*	
C15	0.8733 (3)	0.40755 (10)	−0.04551 (13)	0.0304 (4)	
H15A	1.0034	0.4179	−0.0730	0.036*	
H15B	0.8567	0.4485	−0.0020	0.036*	
C16	0.6690 (3)	0.40310 (10)	−0.12573 (12)	0.0295 (4)	
H16A	0.6455	0.4510	−0.1593	0.035*	
H16B	0.6910	0.3652	−0.1725	0.035*	
C17	0.4681 (3)	0.38384 (10)	−0.08605 (12)	0.0285 (4)	
H17A	0.3395	0.3782	−0.1395	0.034*	
H17B	0.4367	0.4245	−0.0449	0.034*	
C18	0.5024 (3)	0.31257 (10)	−0.02789 (12)	0.0250 (3)	
H18A	0.3726	0.3034	0.0003	0.030*	
H18B	0.5165	0.2709	−0.0707	0.030*	
N1	0.7294 (2)	0.24994 (7)	0.11224 (9)	0.0197 (3)	
N2	0.9074 (2)	0.35684 (7)	0.32526 (10)	0.0219 (3)	
N3	0.8852 (2)	0.05990 (8)	0.12380 (11)	0.0268 (3)	

### Atomic displacement parameters (Å^2^)

**Table:**

	*U*^11^	*U*^22^	*U*^33^	*U*^12^	*U*^13^	*U*^23^
C1	0.0195 (7)	0.0269 (8)	0.0196 (7)	0.0041 (6)	0.0039 (5)	0.0007 (6)
C2	0.0202 (7)	0.0190 (7)	0.0164 (6)	0.0021 (5)	0.0033 (5)	0.0032 (5)
C3	0.0205 (7)	0.0296 (8)	0.0199 (7)	−0.0015 (6)	0.0031 (6)	−0.0010 (6)
C4	0.0231 (7)	0.0326 (9)	0.0235 (7)	0.0012 (6)	0.0089 (6)	0.0022 (7)
C5	0.0319 (8)	0.0269 (8)	0.0231 (7)	0.0061 (7)	0.0114 (6)	−0.0004 (6)
C6	0.0289 (8)	0.0217 (8)	0.0249 (7)	0.0004 (6)	0.0043 (6)	−0.0015 (6)
C7	0.0248 (7)	0.0214 (7)	0.0212 (7)	0.0019 (6)	0.0090 (6)	0.0002 (6)
C8	0.0253 (7)	0.0221 (7)	0.0173 (6)	−0.0018 (6)	0.0049 (6)	−0.0011 (6)
C9	0.0246 (8)	0.0273 (8)	0.0302 (8)	−0.0003 (6)	0.0081 (7)	−0.0021 (7)
C10	0.0311 (8)	0.0324 (9)	0.0317 (9)	−0.0092 (7)	0.0114 (7)	−0.0034 (7)
C11	0.0393 (10)	0.0259 (8)	0.0277 (8)	−0.0097 (7)	0.0048 (7)	0.0014 (7)
C12	0.0336 (9)	0.0242 (9)	0.0384 (10)	0.0013 (7)	0.0072 (8)	0.0044 (8)
C13	0.0198 (7)	0.0208 (7)	0.0187 (6)	0.0015 (6)	0.0049 (5)	−0.0010 (6)
C14	0.0224 (7)	0.0251 (8)	0.0248 (7)	−0.0013 (6)	0.0058 (6)	0.0008 (6)
C15	0.0363 (9)	0.0266 (9)	0.0282 (8)	−0.0056 (7)	0.0065 (7)	0.0020 (7)
C16	0.0395 (10)	0.0269 (8)	0.0211 (7)	0.0012 (7)	0.0041 (7)	0.0029 (6)
C17	0.0291 (8)	0.0307 (8)	0.0234 (7)	0.0062 (7)	0.0002 (6)	0.0014 (7)
C18	0.0205 (7)	0.0295 (8)	0.0238 (7)	0.0003 (6)	0.0016 (6)	0.0027 (6)
N1	0.0226 (6)	0.0211 (6)	0.0157 (6)	0.0025 (5)	0.0048 (5)	−0.0003 (5)
N2	0.0215 (6)	0.0202 (6)	0.0238 (6)	0.0006 (5)	0.0040 (5)	0.0008 (5)
N3	0.0282 (7)	0.0224 (7)	0.0308 (7)	0.0022 (6)	0.0080 (6)	0.0018 (6)

### Geometric parameters (Å, º)

**Table:**

C1—N1	1.4554 (19)	C10—H10	0.9500
C1—C2	1.505 (2)	C11—C12	1.373 (3)
C1—H1A	0.9900	C11—H11	0.9500
C1—H1B	0.9900	C12—N3	1.341 (2)
C2—N2	1.3359 (19)	C12—H12	0.9500
C2—C3	1.390 (2)	C13—N1	1.4821 (19)
C3—C4	1.381 (2)	C13—C18	1.528 (2)
C3—H3	0.9500	C13—C14	1.528 (2)
C4—C5	1.383 (2)	C13—H13	1.0000
C4—H4	0.9500	C14—C15	1.526 (2)
C5—C6	1.380 (2)	C14—H14A	0.9900
C5—H5	0.9500	C14—H14B	0.9900
C6—N2	1.345 (2)	C15—C16	1.527 (2)
C6—H6	0.9500	C15—H15A	0.9900
C7—N1	1.4642 (19)	C15—H15B	0.9900
C7—C8	1.509 (2)	C16—C17	1.520 (3)
C7—H7A	0.9900	C16—H16A	0.9900
C7—H7B	0.9900	C16—H16B	0.9900
C8—N3	1.334 (2)	C17—C18	1.530 (2)
C8—C9	1.394 (2)	C17—H17A	0.9900
C9—C10	1.380 (2)	C17—H17B	0.9900
C9—H9	0.9500	C18—H18A	0.9900
C10—C11	1.383 (3)	C18—H18B	0.9900
			
N1—C1—C2	113.59 (12)	N1—C13—C18	110.69 (13)
N1—C1—H1A	108.8	N1—C13—C14	115.35 (12)
C2—C1—H1A	108.8	C18—C13—C14	110.47 (13)
N1—C1—H1B	108.8	N1—C13—H13	106.6
C2—C1—H1B	108.8	C18—C13—H13	106.6
H1A—C1—H1B	107.7	C14—C13—H13	106.6
N2—C2—C3	122.37 (14)	C15—C14—C13	110.87 (14)
N2—C2—C1	116.00 (13)	C15—C14—H14A	109.5
C3—C2—C1	121.58 (14)	C13—C14—H14A	109.5
C4—C3—C2	119.13 (15)	C15—C14—H14B	109.5
C4—C3—H3	120.4	C13—C14—H14B	109.5
C2—C3—H3	120.4	H14A—C14—H14B	108.1
C3—C4—C5	119.17 (15)	C14—C15—C16	110.99 (14)
C3—C4—H4	120.4	C14—C15—H15A	109.4
C5—C4—H4	120.4	C16—C15—H15A	109.4
C6—C5—C4	117.92 (15)	C14—C15—H15B	109.4
C6—C5—H5	121.0	C16—C15—H15B	109.4
C4—C5—H5	121.0	H15A—C15—H15B	108.0
N2—C6—C5	123.87 (16)	C17—C16—C15	110.62 (14)
N2—C6—H6	118.1	C17—C16—H16A	109.5
C5—C6—H6	118.1	C15—C16—H16A	109.5
N1—C7—C8	111.97 (12)	C17—C16—H16B	109.5
N1—C7—H7A	109.2	C15—C16—H16B	109.5
C8—C7—H7A	109.2	H16A—C16—H16B	108.1
N1—C7—H7B	109.2	C16—C17—C18	111.50 (14)
C8—C7—H7B	109.2	C16—C17—H17A	109.3
H7A—C7—H7B	107.9	C18—C17—H17A	109.3
N3—C8—C9	122.00 (15)	C16—C17—H17B	109.3
N3—C8—C7	116.69 (14)	C18—C17—H17B	109.3
C9—C8—C7	121.29 (14)	H17A—C17—H17B	108.0
C10—C9—C8	119.52 (16)	C13—C18—C17	111.29 (14)
C10—C9—H9	120.2	C13—C18—H18A	109.4
C8—C9—H9	120.2	C17—C18—H18A	109.4
C9—C10—C11	118.68 (17)	C13—C18—H18B	109.4
C9—C10—H10	120.7	C17—C18—H18B	109.4
C11—C10—H10	120.7	H18A—C18—H18B	108.0
C12—C11—C10	118.01 (16)	C1—N1—C7	110.47 (12)
C12—C11—H11	121.0	C1—N1—C13	112.17 (12)
C10—C11—H11	121.0	C7—N1—C13	114.30 (12)
N3—C12—C11	124.36 (17)	C2—N2—C6	117.52 (14)
N3—C12—H12	117.8	C8—N3—C12	117.42 (15)
C11—C12—H12	117.8		
			
N1—C1—C2—N2	−142.02 (14)	C15—C16—C17—C18	−55.53 (19)
N1—C1—C2—C3	40.6 (2)	N1—C13—C18—C17	175.48 (13)
N2—C2—C3—C4	−1.6 (2)	C14—C13—C18—C17	−55.51 (17)
C1—C2—C3—C4	175.56 (14)	C16—C17—C18—C13	55.43 (18)
C2—C3—C4—C5	0.6 (2)	C2—C1—N1—C7	−159.59 (13)
C3—C4—C5—C6	0.6 (2)	C2—C1—N1—C13	71.62 (17)
C4—C5—C6—N2	−0.8 (3)	C8—C7—N1—C1	73.28 (16)
N1—C7—C8—N3	−140.81 (14)	C8—C7—N1—C13	−159.09 (13)
N1—C7—C8—C9	40.9 (2)	C18—C13—N1—C1	−159.74 (12)
N3—C8—C9—C10	1.0 (2)	C14—C13—N1—C1	73.93 (15)
C7—C8—C9—C10	179.14 (15)	C18—C13—N1—C7	73.51 (16)
C8—C9—C10—C11	−0.2 (3)	C14—C13—N1—C7	−52.83 (17)
C9—C10—C11—C12	−0.6 (3)	C3—C2—N2—C6	1.4 (2)
C10—C11—C12—N3	0.7 (3)	C1—C2—N2—C6	−175.90 (14)
N1—C13—C14—C15	−176.97 (13)	C5—C6—N2—C2	−0.2 (2)
C18—C13—C14—C15	56.58 (17)	C9—C8—N3—C12	−0.9 (2)
C13—C14—C15—C16	−57.35 (18)	C7—C8—N3—C12	−179.15 (15)
C14—C15—C16—C17	56.55 (19)	C11—C12—N3—C8	0.1 (3)

### Hydrogen-bond geometry (Å, º)

**Table:**

*D*—H···*A*	*D*—H	H···*A*	*D*···*A*	*D*—H···*A*
C4—H4···N2^i^	0.95	2.55	3.475 (2)	166 (1)
C11—H11···N2^ii^	0.95	2.64	3.511 (2)	153 (1)

Symmetry codes: (i) *x*−1, *y*, *z*; (ii) *x*−1/2, *y*−1/2, *z*.
